# The Impact of Active Workstations on Workplace Productivity and Performance: A Systematic Review

**DOI:** 10.3390/ijerph15030417

**Published:** 2018-02-27

**Authors:** Samson O. Ojo, Daniel P. Bailey, Angel M. Chater, David J. Hewson

**Affiliations:** 1 Institute for Health Research, University of Bedfordshire, Luton LU1 3JU, UK; samson.ojo@study.beds.ac.uk; 2 Institute for Sport and Physical Activity Research, School of Sport Science and Physical Activity, University of Bedfordshire, Bedford MK41 9EA, UK; Daniel.Bailey@beds.ac.uk (D.P.B.); Angel.chater@beds.ac.uk (A.M.C.)

**Keywords:** sedentary behavior, physical activity, sit-stand, active workstation, treadmill desk, productivity, performance

## Abstract

Active workstations have been recommended for reducing sedentary behavior in the workplace. It is important to understand if the use of these workstations has an impact on worker productivity. The aim of this systematic review was to examine the effect of active workstations on workplace productivity and performance. A total of 3303 articles were initially identified by a systematic search and seven articles met eligibility criteria for inclusion. A quality appraisal was conducted to assess risk of bias, confounding, internal and external validity, and reporting. Most of the studies reported cognitive performance as opposed to productivity. Five studies assessed cognitive performance during use of an active workstation, usually in a single session. Sit-stand desks had no detrimental effect on performance, however, some studies with treadmill and cycling workstations identified potential decreases in performance. Many of the studies lacked the power required to achieve statistical significance. Three studies assessed workplace productivity after prolonged use of an active workstation for between 12 and 52 weeks. These studies reported no significant effect on productivity. Active workstations do not appear to decrease workplace performance.

## 1. Introduction

Sedentary behavior can be defined as “any waking behavior characterized by an energy expenditure ≤1.5 metabolic equivalents (METs), while in a sitting, reclining or lying posture” ([1], p. 9). Epidemiological studies have revealed that excessive time spent sitting can increase the likelihood of many health outcomes, including type 2 diabetes, cardiovascular disease, cancer, obesity, and all-cause mortality [2,3,4]. This is usually regardless of the amount of time spent in moderate-to-vigorous physical activity [2,5]. Office workers are at increased risk as they spend more than half of their workday sitting [6,7]. In one study, office based employees spent 82% of working hours and 69% of non-work hours engaged in sedentary behavior [8].

Increasing evidence has shown that sedentary behavior in the workplace can be curtailed by making changes to the work environment, such as the introduction of active workstations [9]. An active workstation enables people to incorporate physical activity into a sedentary task, and can include different types of activity such as walking on a treadmill, pedaling a stationary bicycle, using an elliptical trainer, or simply standing at a height-adjustable desk [10]. For instance, a pilot study replaced “stationary sitting desks” with “sit-stand workstations” to allow office workers the option to alternate between sitting and standing [11]. After one week, the intervention group significantly decreased their sitting time by 143 min per day compared with the control group [11]. A similar study using treadmill desks reported a significant 9% reduction in sedentary time by over 90 min at the end of a six month intervention, but this effect declined to 43 min at 12 months post-intervention [12]. In contrast, during a two-week intervention, the adoption of standing ‘hot’ desks in an open plan office in which office workers were encouraged to stand as often as possible whilst working did not change employees’ sitting time [13]. Portable pedal machines have also been used to increase activity while sitting, which is termed “active sitting” and one study reported a 60 min per day reduction in sedentary time at the end of a three-month intervention [14]. These studies suggest that sitting time can be reduced in the workplace using active workstations. 

While the initiatives outlined above appear to be effective in reducing sitting time, there has been limited research regarding the effect of active workstations on performance and productivity variables [15]. There is a need, therefore, to investigate the effect of active workstations on productivity and performance to identify their suitability for use in the workplace. The term performance, which is often used interchangeably with productivity, is sometimes described as an umbrella terminology for every concept that determines how successful companies are [16]. Performance can also be defined as “the proficiency with which individuals perform the core substantive or technical tasks central to their job” ([17], p. 610). In the present study, performance refers to the efficiency of employees in tasks central to their office work including, but not limited to, data entry, reading and browsing. The term productivity is defined in this review as the quality or state of yielding large result or yielding abundantly, which is often determined by the ratio of output to input [16]. It is inherently complex to determine the productivity of office workers as their activities vary widely, including both repetitive tasks and creativity, depending on the job requirements. For the purposes of this review, worker productivity will include evaluations of work output, as well as evaluations of cognitive function that could be required to carry out office-related tasks [18].

The evidence regarding the effect of active workstations on productivity and performance is equivocal [19]. For instance, in one study in which sit-stand workstations were used, office workers were reported to feel more productive, energized, and focused [7]. In contrast, small non-significant reductions in data entry efficiency and accuracy for a data entry task were found among male university students while standing, when compared to sitting [20]. However, in such studies using simulated workspaces, it is debatable whether the results are applicable to a real office environment. In other studies involving the use of a treadmill desk, walking was identified as a hindrance to mouse-related tasks such as typing, possibly due to such tasks requiring a steady posture and the use of hands for precise execution [21,22]. Given the inconsistency in the current research findings, most managers might be reluctant to implement active workstations in the workplace. It has been suggested in one study that it would be very unlikely for organizational management to institute the use of treadmill desks if productivity is harmed [23]. This uncertainty reinforces the need for further investigation of the effect of active workstations on productivity and performance of workers. The aim of this systematic review was therefore to examine literature investigating the effect of using active workstations on productivity and workplace performance.

## 2. Materials and Methods

### 2.1. Data Sources and Study Selection 

Ethical approval for the systematic review protocol was obtained from the Institute for Health Research Ethics Committee at the University of Bedfordshire on the 28 April 2016 (IHREC611). A systematic literature search was carried out to identify relevant studies. The searched databases were PsycInfo, SPORTDiscus, Web of Science, and PubMed for studies published between January 2005 and December 2016. The 2005 cut-off was chosen as very little literature on active workstations exists before this date. The search terms included “active workstation,” “sit-stand desk” “treadmill workstation”, “treadmill desk”, “workplace”, “work setting”, “productivity” and “performance”. Duplicates were removed before two reviewers (SO and DH) independently screened titles and abstracts of all identified articles. Only studies that were published in peer-reviewed journals were included. Additional relevant studies were sourced manually from the reference lists of the retrieved articles. Studies were eligible for inclusion if they met the criteria stated in Table 1, using the PICO (T) framework [24]. The PRISMA four-phased flow diagram was used in summarizing the study selection processes [25].

### 2.2. Quality Appraisal

The methodological quality of the selected articles was independently assessed by two reviewers (Samson Ojo and Daniel Bailey). Disagreements were resolved with scores from a third reviewer (David Hewson). Eligible studies were assessed with a modified version of the Downs and Black checklist [26] for reporting, internal validity-confounding, internal validity-bias and external validity. The original checklist contains 27 questions, but four questions were considered inapplicable, three of which related to blinding and concealment, which are not relevant in active workstation interventions. A question related to determining power was also omitted. Downs and Black assign two point compliance criteria, giving a maximal score of 24, with the cut-off for inclusion set to 12.

### 2.3. Extraction and Management of Data

Authors of included studies with missing or incomplete data were contacted by email to retrieve further information. In studies where effect sizes were not provided, Cohen’s d, otherwise known as the Standardized Mean Difference (SMD), was calculated to determine the effect of the intervention on performance and productivity. The SMD is calculated by dividing the mean difference by the pooled standard deviation [27]. The scale proposed by Hopkins and colleagues was used to describe the magnitude of the SMD observed [28]. This scale describes effects as “trivial” (<0.2), “small” (0.2 ≤ 0.6), “moderate” (0.6 ≤ 1.2), “large” (1.2 ≤ 2.0) or “very large” (≥2.0). Effect sizes were expressed as negative to indicate decreased performance, irrespective of the direction of the effect. For instance, an increased error rate for a task corresponded to a negative effect size, whereas an increased word count when typing would have given a positive effect size. All reported effect sizes are in comparison with the control or baseline condition.

## 3. Results

### 3.1. Article Selection

**Figure 1:**
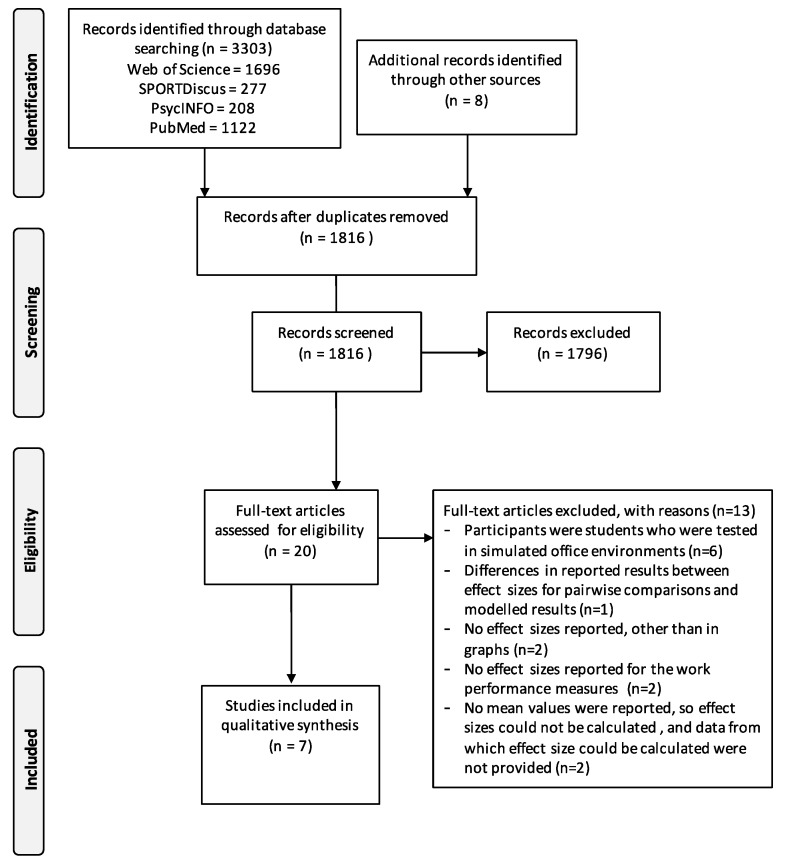
Preferred Reporting Items for Systematic Review and Meta-Analyses (PRISMA) flow chart of study selection [41].

A flow chart of the selection process is shown in Figure 1. The initial search identified a total of 3303 articles, which was reduced to 1826 after duplicates were removed. The titles and abstracts of the remaining articles were screened against the inclusion criteria, with 1796 articles excluded for reasons including relevance, the population studied, and being an exercise or physical activity intervention rather than an intervention targeting sedentary behavior. Twenty articles were identified as potentially relevant and assessed for eligibility. Thirteen articles were rejected after full-text screening as some of these studies did not report effect sizes or data to calculate effect size, and six studies used students working in simulated office environments. The resulting sample consisted of seven articles, with no additional studies identified following a search through the references of the included articles [12,29,30,31,32,33,34,35,36,37,38,39,40].

### 3.2. Study Characteristics

All included studies used office workers as participants [12,31,32,34,37,38,40]. The articles contained three different intervention types including treadmill desks [12,37], cycling workstations [34,40], and sit-stand workstations [31,32,37,38]. A total of 16 different productivity and work performance outcomes were identified. To this end, it was deemed that a meta-analysis would not be appropriate given the diversity of the outcome measures and study designs. Detailed characteristics of the selected studies including quality appraisal scores are shown in Table 2. Six of the studies reported details of ethical approval. The authors of the remaining study were contacted by email and confirmed details of their ethical approval.

### 3.3. Cognitive Performance: A Measure of Productivity

The majority of studies presented cognitive performance as outcome measures for productivity. Cognitive performance was assessed in five of seven studies using a variety of tests [32,34,37,38,40]. These tests have been classified into the following categories depending on the element of cognitive function being assessed: attention, memory, and reasoning. All cognitive function changes were made in comparison to a control condition of sitting.

#### 3.3.1. Attention

Three studies assessed attention responses when using active workstations (either treadmill, cycling or sit-stand desks) [37,38,40], with the results shown in Table 3. With respect to standing workstations, all differences observed were trivial. When participants used a cycling workstation, there were 12 different attention tests used, with most of these showing no difference in attention, or a small improvement [40]. Only one test of attention was reported while walking using a treadmill desk with a trivial increase in attention reported when compared with sitting [37].

#### 3.3.2. Memory

Three studies examined memory performance in response to active workstation use with results shown in Table 4 [37,38,40]. In two studies, a trivial increase in memory performance was observed using both a sit-stand workstation and treadmill desk [37,38]. However, in the remaining study, memory performance was decreased when using a cycling workstation [40]. The decreases observed in this study were trivial, regardless of the component of the auditory verbal learning test used, but none of these differences were statistically significant.

#### 3.3.3. Reasoning and Reaction Time

One study investigated reasoning responses [34] and another reaction times [38], with the results of both studies shown in Table 5. None of the differences in reasoning responses between a cycling workstation and sitting were significant. No significant differences in reaction time were found when using a sit-stand workstation, compared to sitting.

### 3.4. Work-Related Performance

Two different types of work-related performance (typing and proof reading task) were assessed. The results of using active workstations on work-related performance tests are shown in Table 6. Two studies examined the effect of using a cycling workstation on typing performance [34,40] and one study evaluated proofreading performance when using a sit-stand desk [35]. With respect to typing, the only significant changes reported were in the study by Koren et al. [34], in which a small decrease in performance was observed. However, only a trivial decrease in performance was reported by Torbynes et al. [40]. Trivial increases were recorded for the proof reading performance, although no significant difference was observed [38].

### 3.5. Productivity after Prolonged Use of Active Workstations

Three studies assessed workplace productivity after prolonged use of sit-stand workstations [12,31,32] through the use of the Brickencamp d2 test to examine concentration performance [32], monitoring average call handling time, hold time on a call, talk time and wrap up time on a call [31], and through employee- and supervisor-rated performance [12]. In the study by Donath et al. [32], participants used the workstation for 12 weeks, while Chau et al. [31] assessed productivity after 19 weeks of use, and Koepp et al. [12] after one year. The results obtained from all three studies are shown in Table 7. No significant differences were observed in response to any of the interventions, although some of the outcomes measured did have moderate changes in productivity.

## 4. Discussion

The aim of this review was to determine whether using an active workstation had any effect on productivity or workplace performance. The seven studies reviewed fell into two distinct categories with respect to the methods used to assess both productivity and performance. Most of the studies estimated productivity based on cognitive performance tests using laboratory-based and/or simulated-office tasks as outcome measures, while work performance was estimated by typing and proofreading. Four studies evaluated productivity and work-related performance while using an active workstation, whereas the other three studies assessed workplace productivity after prolonged use of active workstations.

The studies examining cognitive performance as a measure of productivity used a range of tests to assess different elements of cognition, including attention, memory, reasoning, and reaction time. With respect to attention, both of the studies reported only trivial effects when using a sit-stand workstation [37,38]. Similar results were reported in the studies in which attention was measured while using a cycling or walking workstation, with most tests producing trivial differences [37,40]. The results of the studies in which memory was assessed while using an active workstation followed the same pattern with use of three types of workstations leading to trivial increases in memory that were non-significant [37,38,40].

It appears that using a sit-stand workstation has no effect on productivity when the person is standing, indicating that alternating between standing and sitting may not have any detrimental effect on the amount and quality of work being produced. A lack of significantly different results were observed for both cycling and walking workstations, which could be an indication that these two workstations may not pose any threat to the quality of work produced, although it is worth noting that several effects that could be considered moderate using the Hopkins’ scale were not detected as statistically significant, perhaps owing to low power in the studies [28]. It should also be noted that participants lacked familiarity with the active workstations used in most of the studies, so work productivity and performance could be expected to improve with habitual use. It is also possible that any potentially beneficial effects of long-term use of active workstations would not have been observed given the short time in which participants used the workstations. It has been suggested that using an active workstation could influence long-term performance and workplace productivity. Only three studies assessed workplace productivity after prolonged use of active workstations, with the duration of these studies ranging from 12–52 weeks [12,31,32]. None of these studies reported any change in productivity after long-term use of an active workstation. However, as with the short-term studies assessing productivity responses, two studies [34,40] had relatively low power but with effect sizes as large as 1.8. This indicates a large effect that was not found to be statistically significant [28]. Future research in this area needs to be carried out with sufficient power to investigate the exact impact of short-term use of active workstations on productivity.

Based on the findings of this review, it appears that there is insufficient evidence regarding the effect of active workstations on productivity and workplace performance. The studies reviewed fell into two categories and either focused on cognitive performance while using an active workstation that participants were not familiar with, or they were long-duration studies in which productivity was measured using simple tools such as self-rated questionnaires and call handling time. Future research should investigate the effect of active workstations on productivity, making sure to use non-subjective measures of productivity.

The potential of active workstations to reduce the amount of sedentary behavior in the workplace was the focus of another recent systematic review. In this review, Chu and colleagues [42] reported that sit-stand workstations were effective in reducing sitting time, although not as effective as multi-component interventions. However, this review did not examine whether the use of active workstations had any effect on productivity or workplace performance. In another systematic review, MacEwen and colleagues [43] looked at the effect of sit-stand and treadmill desks on both physiological and psychological outcomes. The psychological outcomes included both typing and mouse clicking performance. They reported no change in work performance when using a standing workstation, but a decrease in performance when using a treadmill desk that was proportional to the speed at which the participants were walking. The results of this present review are consistent with the review of MacEwen and colleagues [43], in which typing task performance had a large decrease when cycling [34]. The magnitude of the decrease in performance could be attributed to the intensity of the activity [44]. A similar systematic review by Cao and colleagues [44] examined the effect of active workstations on both energy expenditure and work performance. They evaluated performance when using a treadmill desk, with decreased performance in typing tasks, mouse clicking, and transcribing speed. However, none of the articles included were longitudinal studies in which changes in performance were evaluated over time. Likewise, Commissaris and colleagues [45] evaluated the effect of workplace interventions to reduce sedentary behavior on physical activity levels and productive work. They reported conflicting evidence for the effects of active workstations on work performance, however, most studies were of short duration, with performance assessed using self-reported performance measures. 

The key finding of the present study was that sit-stand workstations do not appear to significantly decrease performance, which contradicts a potential concern of employers [46]. In fact, in some cases active workstations might enhance employee performance and productivity. However, although treadmill and cycling workstations might decrease both productivity and performance, inappropriate study design, including small sample size and lack of familiarity with the workstations, meant that a true reflection of their impact could not be determined. The articles included in this systematic review were limited to those from peer-reviewed journals, thus excluding other studies such as unpublished papers, dissertations and theses. Although this might have introduced selection bias, it also ensured that the sources selected were of sufficient quality. In addition, further research is needed to identify the most appropriate tools to quantify work productivity and workplace performance. It has been suggested that the Work Limitations Questionnaire (WLQ) [47] is the most suitable for research use when considering the effect of physical activity [48]. The WLQ provides subjective measures of both productivity and presenteeism [49]. An alternative measure of productivity could be ecological momentary assessment (EMA), which has been used in a variety of different contexts [50]. The EMA technique involves participants being prompted in their normal working environment at random times throughout the day to respond about their current behavior and symptoms, which has the advantage of sampling as close as possible to the event, thus limiting recall bias [51]. This technique is being increasingly used due to the availability of electronic devices such as smartphones, which can be used to deliver the prompts at random time points throughout a working day, so can easily be adapted to an office environment.

## 5. Conclusions

This systematic review was undertaken to identify whether active workstations had any effect on productivity or workplace performance. Most studies evaluated productivity and work performance during single-session trials with the evidence suggesting that sit-stand workstations have no detrimental effect on these outcomes. Limited evidence was found to suggest that treadmill and cycling workstations might decrease some aspects of productivity and performance, but this could be due to a lack of familiarity with the workstations. In the remaining studies in which the long-term use of active workstations was examined, the tools used to assess productivity and work performance were inadequate. Future studies should investigate the impact of active workstations on employees’ productivity and work performance in the workplace. 

## Figures and Tables

**Table 1:** Inclusion and exclusion criteria.

Term	Inclusion Description	Exclusion Description
Population	Healthy, working age, adult employees (≥18 years old) from developed countries	Studies where recruited participants have specific comorbidities or diseases (such as diabetes, arthritis, cancer, stroke), special populations (pregnant, physical disability, or cognitive disability), or targeted pain management or musculoskeletal issues
		Non-employees (students) in an office-simulated environment
Intervention	Use of workstations such as sit-stand desk, treadmill desk; cycling desk	Not office based, not workstations
Comparison	Any comparative study with either baseline measures or non-intervention group as control for comparison.	No comparison measures
Outcomes	Productivity or work performance	No measure of productivity or work performance
Trial design	Randomized controlled trials or quasi-experimental trials	Observational studies

**Table 2:** Characteristics of the selected articles.

Authors	Participants	Study Design and Intervention	Performance Measures	Quality (Max 24)
Chau et al. (2016) [31] Australia Call center staff	14 females, 17 males 33.0 ± 10.8 years, unspecified health status BMI 26.8 ± 5.5 kg/m^2^	Quasi-experimental trial 19-week intervention Sit-stand workstations	Work performance Call handling time Time on call Hold time on call Wrap up time on call Customer rating	13
Donath et al. (2015) [32] Switzerland	23 females, 8 males 42.4 ± 11.0 years, Healthy office workers BMI 24.2 ± 4.4 kg/m^2^	Single-blinded RCT 12-week intervention Sit-stand workstation	Cognition performance: Attention (Brickenkamp d2)	19
Koepp et al. (2013) [12] USA	25 females, 11 males 42 ± 10 years, Office workers able to walk at 3 mph for 30 min, not pregnant BMI 29 ± 7 kg/m^2^	Prospective trial 1-year intervention Treadmill desk	Work performance Employee-rated performance Supervisor-rated performance	15
Koren et al. (2016) [34] Slovenia	13 participants but no. of females and males not specified 30.6 ± 3.8 years, healthy office workers BMI 21.2 ± 12.0 kg/m^2^	Crossover design 30-min intervention with two exercise intensities Cycling workstation	Cognitive performance Reasoning (Wonderlic test) Work performance Typing speed and error rate	17
Ohlinger et al. (2011) [37] USA	Unreported no of females and males 43.2 ± 9.3 years, university ≤ 150 kg, walk unaided BMI 28.5 ± 5.9 kg/m^2^	Quasi-experimental trial 75-min intervention Treadmill desk Sit-stand workstation	Cognitive performance Attention (Stroop) Memory (Auditory Consonant Trigram)	14
Russell et al. (2015) [38] Australia	26 females, 10 males 40.1 ± 11.9 years, university employees unreported health status Unreported BMI	RCT Two-week intervention Sit-stand workstation	Cognitive performance Attention (Stroop) Memory (Digit Span) Reaction Time (Digit Symbol Coding) Work performance Proof reading (speed and error rate)	17
Torbeyns et al. (2016) [40] Belgium	16 females, 7 males 35.7 ± 10.3 years, Healthy office workers BMI 23.2 ± 3.0 kg/m^2^	Quasi-experimental trial 30-min intervention Cycling workstation	Cognitive performance Attention (Stroop, Rosvold) Memory (Rey Auditory Verbal Learning) Work performance Typing speed and error rate	17

Quality measured using modified Downs and Black checklist; Data reported to 1 significant figure where authors included sufficient precision. BMI: Body Mass Index.

**Table 3:** Effect of active workstation use on attention.

Condition	Author	Performance Test	*n*	SMD	Effect Size Magnitude
Standing	Ohlinger et al. (2011) [37]	Stroop colour word test (T-score—number of correct items)	50	0.02	Trivial decrease
	Russell et al. (2015) [38]	Choice Reaction Time (ms)	36	0.06	Trivial increase
		Choice Reaction Time accuracy (%)	36	0.02	Trivial increase
		Stroop incongrunet (s)	36	0.06	Trivial decrease
Cycling	Torbeyns et al., 2016 [40]	Rosvold continuous performance test reaction time (ms)	23	0.73	Moderate increase *
		Rosvold continuous performance test accuracy (%)	23	1.00	Moderate decrease
		Stroop accuracy color congruent stimuli (%)	23	0.00	Trivial—no change
		Stroop accuracy color incongruent stimuli (%)	23	0.06	Trivial decrease
		Stroop accuracy neutral stimuli (%)	23	0.03	Trivial decrease
		Stroop accuracy word congruent stimuli (%)	23	0.06	Trivial increase
		Stroop accuracy word incongruent stimuli (%)	23	0.02	Trivial increase
		Stroop reaction time color congruent stimuli (ms)	23	0.20	Small increase
		Stroop reaction time color incongruent stimuli (ms)	23	0.09	Trivial increase
		Stroop reaction time neutral stimuli (ms)	23	0.18	Trivial increase
		Stroop reaction time word congruent stimuli (ms)	23	0.21	Small increase
		Stroop reaction time word incongruent stimuli (ms)	23	0.34	Small increase
Walking	Ohlinger et al., 2011 [37]	Stroop color word test (T-score—number of correct items)	50	0.03	Trivial increase

* Significantly different from sitting condition. SMD: Standardized Mean Difference.

**Table 4:** Effect of active workstation use on memory.

Condition	Author	Performance Test	*n*	SMD	Effect Size Magnitude
Standing	Ohlinger et al., 2011 [37]	Auditory consonant trigram test (number of correct consonants)	50	0.11	Trivial increase
	Russell et al., 2015 [38]	Digit Span subtest—number correct backwards	36	0.11	Trivial increase
		Digit Span subtest—number correct forwards	36	0.13	Trivial increase
		Letter number sequencing test	36	0.19	Trivial increase
Cycling	Torbeyns et al., 2016 [40]	Rey Auditory Verbal Learning Test (Correctly recognized words)	23	0.15	Trivial decrease
		Rey Auditory Verbal Learning Test (Incorrectly recognized words)	23	0.00	Trivial—no change
		Rey Auditory Verbal Learning Test (Recalled words)	23	0.13	Trivial decrease
		Rey Auditory Verbal Learning Test (Repeated words)	23	0.12	Trivial increase
Walking	Ohlinger et al., 2011 [37]	Auditory consonant trigram test (number of correct consonants)	50	0.06	Trivial increase

**Table 5:** Effect of active workstation use on reasoning and reaction time.

Condition	Author	Performance Test	*n*	SMD	Effect Size Magnitude
Cycling	Koren et al., 2016 [34]	Reasoning: Wonderlic test score (40 W workload)	13	0.13	Trivial increase
		Reasoning: Wonderlic test score (80 W workload)	13	0.25	Small decrease
		Reasoning: Wonderlic test time (s) (40 W workload)	13	0.05	Trivial decrease
		Reasoning: Wonderlic test time (s) (80 W workload)	13	0.52	Small increase
Standing	Russell et al. (2015) [38]	Reaction time: Digit Symbol Coding subtest (total)	36	0.02	Trivial decrease
		Reaction time: Trail making test (s)	36	0.09	Trivial decrease

**Table 6:** Effect of active workstation use on work-related performance tasks.

Condition	Author	Performance Test	*n*	SMD	Effect Size Magnitude
Standing	Russell et al. (2015) [38]	Proof reading task (errors identified)	36	0.03	Trivial increase
		Proof reading task (time)	36	0.11	Trivial increase
Cycling	Torbeyns et al. (2016) [40]	Typing test (net words per min)	23	0.05	Trivial decrease
		Typing time (s) (40 W workload)	13	0.51	Small decrease *
	Koren et al. (2016) [34]	Typing time (s) (80 W workload)	13	0.58	Small decrease *
		Typing errors (number) (40 W workload)	13	1.66	Large decrease
		Typing errors (number) (80 W workload)	13	1.81	Large decrease

* Significantly different from sitting condition.

**Table 7:** Effect of active workstation use on work-related productivity tasks.

Condition	Author	Performance Test	Trial Duration	*n*	SMD	Effect Size Magnitude
Standing	Donath et al. (2015) [32]	Brickencamp d2 test (% correct, 3 prompts/day)	12 weeks	15	0.37	Small increase
		Brickencamp d2 test (net performance, 3 prompts/day)	12 weeks	15	0.46	Small increase
		Brickencamp d2 test (% correct, no prompt)	12 weeks	16	0.45	Small increase
		Brickencamp d2 test (net performance, no prompt))	12 weeks	16	0.69	Moderate increase
	Chau et al. (2016) [31]	Average call handling time (min)	19 weeks	16	0.33	Small decrease
		Customer rating	19 weeks	16	0.16	Trivial increase
		Hold time on call (min)	19 weeks	16	0.60	Moderate decrease
		Talk time on call (min)	19 weeks	16	0.05	Trivial increase
		Wrap up time on call (min)	19 weeks	16	0.20	Small increase
Walking	Koepp et al. (2013) [12]	Employee-rated performance (weekly survey)—overall	1 year	23	0.04	Trivial decrease
		Employee-rated performance (weekly survey)—overall	1 year	13	0.22	Trivial decrease
		Employee-rated performance (weekly survey)—quality	1 year	13	0.05	Trivial increase
		Employee-rated performance (weekly survey)—quality	1 year	23	0.37	Small decrease
		Employee-rated performance (weekly survey)—quantity	1 year	23	0.13	Trivial decrease
		Employee-rated performance (weekly survey)—quantity	1 year	13	0.24	Small increase
		Employee-rated performance (weekly survey)—interaction	1 year	13	0.04	Trivial decrease
		Employee-rated performance (weekly survey)—interaction	1 year	23	0.33	Small decrease
		Supervisor-rated performance (weekly survey)—overall	1 year	23	0.35	Small decrease
		Supervisor-rated performance (weekly survey)—overall	1 year	13	0.60	Moderate decrease
		Supervisor-rated performance (weekly survey)—quality	1 year	13	0.15	Trivial decrease
		Supervisor-rated performance (weekly survey)—quality	1 year	23	0.31	Small decrease
		Supervisor-rated performance (weekly survey)—quantity	1 year	13	0.18	Trivial decrease
		Supervisor-rated performance (weekly survey)—quantity	1 year	23	0.26	Small decrease
		Supervisor-rated performance (weekly survey)—interaction	1 year	23	0.05	Trivial decrease
		Supervisor-rated performance (weekly survey)—interaction	1 year	13	0.15	Trivial decrease

Brickencamp d2 test evaluates concentration.
